# Antimicrobial and prebiotic activity of mannoproteins isolated from conventional and nonconventional yeast species—the study on selected microorganisms

**DOI:** 10.1007/s11274-022-03448-5

**Published:** 2022-11-02

**Authors:** Anna Bzducha Wróbel, Pavol Farkaš, Paulina Chraniuk, Dominika Popielarz, Alicja Synowiec, Katarzyna Pobiega, Monika Janowicz

**Affiliations:** 1 Department of Food Biotechnology and Microbiology, Institute of Food Sciences, Warsaw University of Life Sciences, Nowoursynowska 159C Street, 02-787 Warsaw, Poland; 2 Department of Immunochemistry of Glycoconjugates, Institute of Chemistry, Slovak Academy of Sciences, Dúbravská Cesta 9, 845 38 Bratislava, Slovakia; 3 Department of Food Engineering and Process Management, Institute of Food Sciences, Warsaw University of Life Sciences-SGGW, Nowoursynowska Str 159C, 02-776 Warsaw, Poland

**Keywords:** Antimicrobial activity, Mannoproteins, *Metschnikowia reukaufii*, Prebiotic, *Saccharomyces cerevisiae*, *Wickerhamomyces anomalus*

## Abstract

Yeast mannoproteins are proposed as a paraprobiotics with antimicrobial and prebiotic properties. They can be used as biopreservatives in food and in diseases therapies. The knowledge about the specificity and/or capability of their influence on the growth of different microorganism is limited. The study determined the effect of mannoprotein preparations of *Saccharomyces cerevisiae* (*S. cerevisiae*) ATCC 7090 and nonconventional yeast origin [*Metschnikowia reukaufii (M. reukaufii)* WLP 4650 and *Wickerhamomyces anomalus (W. anomalus)* CCY 38-1-13] on the growth of selected bacteria of the genera: *Lactobacilllus*, *Limosilatobacillus*, *Limosilatobacillus*, *Bifidobacterium*, *Staphylococcus*, *Enterococcus*, *Pseudomonas, Escherichia*, *Proteus* and *Salmonella*. The degree of stimulation or growth inhibition of tested bacteria depended on the type and dose of the mannoprotein and the bacterial strain. The addition of the tested preparations in the entire range of applied concentrations had a positive effect especially on the growth of *Lactobacillus arabinosus* ATCC 8014 and *Bifidobacterium animalis* subsp. *lactis* B12. Mannoproteins isolated from *S. cerevisiae* limited the growth of the *Escherichia coli (E. coli)* ATCC 25922, *Pseudomonas aureoginosa (P. aureoginosa)* ATCC 27853, *Proteus mirabilis* ATCC 35659 and *Salmonella* Enteritidis ATCC 13076 to the greatest extent, while preparations of *M. reukaufii* and *W. anomalus* origin most effectively limited the growth of *Staphylococcus aureus* strains, *E. coli* and *P. aureoginosa*. The growth of *Enterococcus faecalis* was stimulated by the presence of all studied preparations in most of the concentrations used. Further research will determine how the purification process of studied mannoproteins or oligosaccharide fractions, its structure and composition influence on the growth of selected bacteria and what is the mechanism of its activity.

## Introduction

World Health Organization stimulates the research on prevention and treatment of diseases using therapies other than antibiotics (Tiago et al. 2012). This results from the development and spread of antibiotic resistance of pathogenic microorganisms and occurence of drug residues in the environment and food chain, endangering human and animal health and safety (Valáriková et al., 2020). Natural medicines, probiotics, and some other natural products, including derivatives of yeast cell wall components, may be used for infectious treatment as an antibiotic substitutes (Chen et al. 2021; Liu et al. 2021).

In food industry there has been an increasing interest in the use of natural substances as biopreservatives, including polysaccharides and oligosaccharides with proper structural features that modulate their biological activities (Barreteau et al. 2006). Selective effect of certain insoluble carbohydrates and glycoproteins in inhibiting pathogens and saprofits or stimulating the growth of beneficial microbiota in food production is not well defined (Fernandez et al. 2002; Liu et al. 2021). Mannoproteins are easily available biomaterial isolated from yeast biomass and their synthesis may be based on valorization of a wide range of low-cost agriculture by-products, including lignocellulotic biomass (Klis et al. 2002; Øverland and Skrede 2016; Bzducha-Wróbel et al. 2018a, b; Valáriková et al. 2020; Agboola et al. 2021; Liu et al. 2021). This prompts the implementation of research defining mannoproteins’ functional properties.

Yeast biomass and yeast cell wall products (cell wall preparations, mannoproteins, mannan or *β*-glucan preparations) are used as promoters in animals’ nutrition inter alia due to their capacity to bind enteropathogenic bacteria with positive effect on lactobacilli and bifidobacteria at the same time (Quirós et al. 2011; Øverland and Skrede 2016; Santoviato et al. 2019; Agboola et al. 2021; Liu et al. 2021). Their antimicrobial properties may be improved by chemical modification (Valáriková et al. 2020; Liu et al. 2021). However, only limited data are avialable considering antimicrobial and prebiotic activity of yeast origin preparations (Santovito et al. 2019; Valáriková et al. 2020; Baumgärtner et al. 2022). At the same time, these data apply in particular to preparations derived from *Saccharomyces* yeasts or *Candida* yeasts. There is only a few information about the biological activity of preparations obtained from unconventional yeasts (Tang et al. 2022). Additionally, the chemical structure and composition of yeast cell wall components vary considerably among yeast species, strain, and yeast growth conditions (Lesage and Buseey 2006) determining biological properties of discussed substances (Agboola et al. 2021; Liu et al. 2021). Therefore the effect of yeast cell wall components on microbial growth can not be generalized and needs to be studied to confirm their effectiveness depending on yeast origin.

Appropriate supplementation of probiotics and prebiotics, eg. yeast origin mannoptoteins, mannan-oligosaccharides and *β*-(1,3/1,6)-glucans, may modulate gut microbiota composition, positively affecting different aspects of human and animal health, including prevention of pathogenic infections or even modulation of psychiatric disorders (Calgaro et al. 2021; Baumgärtner et al. 2022; Tang et al. 2022). Therefore, the search for new sources of compounds with prebiotic properties is a current research problem to enhance the activity of probiotic microorganisms appropriate for special therapies.

Presented study was the first step approach to determine the effect of mannoprotein preparations isolated from the biomass of the conventional *Saccharomyces cerevisiae (S. cerevisiae)* ATCC 7090 yeast and the unconventional yeast species, like *Metschnikowia reukaufii (M. reukaufii)* WLP 4650 and *Wickerhamomyces anomalus (W. anomalus)* CCY 38-1-13, on the growth of selected bacteria, such as: beneficial lactic acid bacteria from the genera *Lactobacilllus*, *Limosilatobacillus* and *Limosilatobacillus*, bifidobacteria, as well as pathogenic and saprophytic bacteria of the genera *Staphylococcus*, *Enterococcus*, *Pseudomonas, Escherichia*, *Proteus* and *Salmonella*.

## Materials and methods

### Yeast strains and conditions of yeast cultivation

The biological material that was used to isolate mannopropteins fraction were the conventional yeast *S. cerevisiae* ATCC 7090, and the two strains of unconventional yeast: *M. reukaufii* WLP 4650 and *W. anomalus* CCY 38-1-13. Yeast strains are available at the Museum of Pure Cultures of the Department of Food Biotechnology and Microbiology, Institute of Food Science, Warsaw University of Life Sciences. The yeast was grown in the YPG medium composed with [g/L]: 20 g of peptone, 20 g of glucose and 10 g of yeast extract, and the pH approx. 5.2. The medium (90 mL) was placed in flasks (500 mL) and then sterilized at 121 °C for 20 min. The yeast inoculum was prepared by inoculating the YPG medium with cells collected from the slant. Inoclulum cultivation was carried at 28 °C for 24 h at with a shaker rotation speed of 200 rpm (Control Buechler SM-30 shaker, Germany). The yeast cultures were started by adding 10 mL of yeast inoculum to the 90 mL of medium. Biomass propagation was performed for 48 h under conditions identical to the inoculum culture. A total of a minimum of 60 cultures were performed for each yeast strain. The yeast biomass was collected by culture centrifugation (4500 rpm, 6 min, Hettich zentrifugen Roto Silenta 630 RS), after which the obtained biomass pellet was washed twice with water, each time swirling.

### Isolation of mannoproteins

**Fig. 1:**
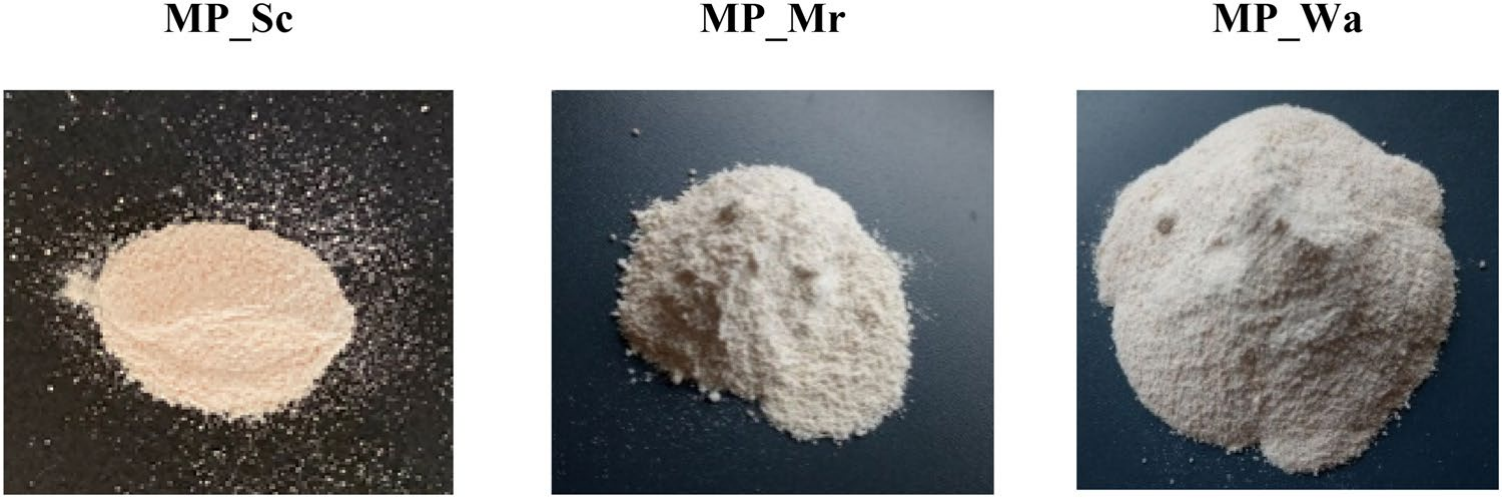
Mannoprotein preparations isolated from the yeast biomass of *S. cerevisiae* ATCC 7090 (MP_Sc), *Metschnikiwia reukaufii* WLP 4650 (MP_Mr) and *W. anomalus* CCY 38-1-13 (MP_Wa)

The proces of mannoproteins isolation from yeast biomass was carried out in accordance with the methodology proposed by Walencka et al. (2007). Yeast biomass was suspended in a buffer composed of 0.1 M potassium citrate and 0.02 M metabisulfate at pH 7.0 to obtain a mixture with a concentration of 20 mass % of biomass in sample. Biomass suspensions in buffer were then autoclaved at 121 °C for 2 h (autoclave PHCBI MLS 3751L, PHC Corporation). After this time, the samples were cooled and centrifuged (5000 rpm,10 min, Centrifuge 5804R, Eppendorf). Then the mixture of 98 vol. % ethanol containing 1 vol. % acetic acid was added to the recovered supernatants and incubated at 4 °C for 24 h to precipitate mannoproteins. The suspension was then centrifuged at 6000 rpm, 15 min (Centrifuge 5804R, Eppendorf). The pellet was dissolved in water and the samples prepared in this way were dialyzed for 24 h using cellulose tubing bags for dialysis (Sigma–Aldrich, St. Louis, USA). The samples were then pre-frozen in an Irinox MOD.51.20 shock cooler (Italy) at an air temperature of − 40 °C and stored under freezing conditions for 24 h, and lyophilized in next step using Christ LCG Gamma 1–16 LSC (United Kingdom) at 63 Pa at the plate temperature of 20 °C. The freeze-dried preparation was ground using a Fritsch Analysette 3 Spartan ball mill. The preparations obtained in this way were stored in tightly closed containers at refrigerated temperature. The photographs of the preparations were shows on Fig. 1.

### Characteristics of mannoprotein preparations

The obtained mannoprotein preparations were characterized considering elemental analysis, total sugar and protein content, determination of molecular mass and spectral analysis using FTIR-ATR and NMR. Elemental analysis was performed using FLASH 2000 Organic elemental analyzer (CHNS-O), Thermo Fisher Scientific. Total sugar content was determined by spectrophotometric method with 3,5-dinitrosalicylic acid (DNS) after acid hydrolysis of the preparations in the environment of 13.5 M H_2_SO_4_ according to Bzducha-Wróbel et al. (2018a, b) (a). To calculate the total sugar content, a standard curve for mannose was used (*y* = 3.612*x*–0.1151, *R*^2^ = 0.9997). The nitrogen content in the preparations was determined by the Kjeldahl method after mineralization of the samples using concentrated acid H_2_SO_4_ and Kjeltabs CT/3.5 catalyst. The amount of titrated nitrogen (TitroLine 5000 apparatus, SI Analytics) was calculated into the protein content using the conversion factor 6.25. Determination of the amino acids composition in obtained preparations was performed using an AAA 500 automatic amino acid analyzer (Ingos), in which the separation was based on ion exchange chromatography. The methodology was based on the procedure described by Szkudzińska et al. (2017).

Analysis of the molecular weight and its distribution based on GPC-HPLC were determined on Accela UHPLC system (Thermo Fisher Scientific Inc.; Waltham, MA, USA) equipped with Accela 1250 Pump and Thermo Scientific Dionex UltiMate™ 3000 fluorescence detector FLD-3100 operating at excitation wavelength of 319 nm and emission wavelength of 419 nm. Two HEMABIO 300 and 1000 columns (8 mm × 250 mm) connected in series were used for analysis. The mobile phase used was 0.1 M NaNO_3_. A set of dextrans (American Polymer Standard Corporation, Mentor, OH, USA) were used for calibration of the HPLC system.

FT-IR spectra were measured on NICOLET Magna 6700 (Thermo Fisher Scientific, USA) spectrometer with DTGS detector, experimental accessory—Smart Orbit and OMNIC 8.0 software was used. Infrared spectral analyses were carried out in mid-infrared region (from 4000 to 400 cm^−1^) and spectral data obtained were presented as absorbance values. Number of scans was set to 128. Diamond Smart Orbit ATR accessory was used for measurements in solid state.

^1^H NMR spectra were acquired in D_2_O (99.97% D) at 45 °C on a Bruker AVANCE III HD X 600 MHz spectrometer (Bruker TopSpin, Rheinstetten, Germany) equipped with a triple inverse TCI H-C/N-D-05-Z liquid He-cooled cryoprobe and processed using Top Spin 4.1.4 software (Bruker). The ^1^H signal of acetone (2.217 ppm) was used as a reference for chemical shifts.

### Determination of the effect of isolated mannoprotein preparations on the growth of selected pathogenic and saprophytic bacteria

This stage of the work studied the effect of the obtained mannoprotein preparations on the growth of the following bacterial strains: *Staphylococcus aureus (S.* *aureus)* ATCC 25923, *S. aureus* ATCC 29213, *Staphylococcus epidermidis (S. epidermidis)* ATCC 12228, *Enterococcus faecalis (E. faecalis)* ATCC 29212, *Pseudomonas aeruginosa (P. aeruginosa)* ATCC 27853, *Proteus mirabilis (P. mirabilis)* ATCC 35659, *P. mirabilis* PCM 543, *Salmonella* Enteritidis (*S.* Enteritidis) ATCC 13076 and *Escherichia coli (E. coli)* ATCC 25922. The strains were obtained from the Museum of Pure Cultures, Department of Biotechnology and Food Microbiology, Institute of Food Sciences, Warsaw University of Life Sciences. The modified procedure for estimation of minimal inhibitory concentration (MIC) was used to study the effect of tested mannoproteins on the bacterial growth. The solution used made it possible to avoid large jumps in the applied concentrations of tested preparations, at the same time allowing to observe the effect of the preparation with smaller changes in concentration. In original MIC procedure the concentration of the active substance is halved in subsequent dilutions.

Bacterial cultivation was carried out in Muller-Hinton Broth (Oxoid) medium with the mass addition of 0% (control), 2%, 3%, 4%, 5%, 6% of mannoprotein preparations obtained from the biomass of the tested conventional and unconventional yeast. All media were autoclaved at 121 °C for 20 min (PHCBI MLS 3751L autoclave, PHC Corporation). The media was then applied under sterile conditions to a 96-well microtiter plate in a volume of 150 μL, and an inoculum of individual bacteria cells was inoculated in a volume of 10 μL per well. The bacterial inoculum for each strain used to inoculate the cultures was prepared by taking the bacterial cells with a loop from 24-h agar cultures and suspending them in physiological saline. The optical density of the slurry was adjusted to 0.5 on the McFarland scale (Densimat, Biomerieux). The inoculated microtiter plates were placed in a Q-CELL 240 incubator where they were incubated for 24 h at 37 °C. The growth results of the tested bacteria were read using Mulitiskan Sky apparatus (ThermoFisher Scientific) by measuring the optical density of cultures at a wavelength of 590 nm at time 0 (immediately after inoculation) and after 24 h of cultivation. The growth intensity of the tested bacteria was determined on the basis of changes in the optical density of the cultures relating them to the changes in the control medium, which were treated as growth under optimal conditions (100%).

### Determination of the prebiotic properties of the obtained mannoprotein preparations

The prebiotic activity of the obtained preparations was determined in relation to the cultures of lactic bacteria and bifidobacteria. The following strains of bacteria were tested: *Lactobacillus arabinosus* ATCC 8014, *Levilactobacillus brevis (L. brevis)* DSMZ 20053, *Limosilactobacillus fermentum (L. fermentum)* KKP 809, *Bifidobacterium bifidum (B. bifidum)* Bb2 and *Bifidobacterium animalis (B. animalis)* subsp. *lactis* B12. Microorganisms were obtained from the Museum of Pure Cultures, Department of Biotechnology and Food Microbiology, Institute of Food Sciences, Warsaw University of Life Sciences. The research was carried out in a microculture system with the use of 96-well microtiter plates. Bacteria were grown in MRS medium (Oxoid) with the mass addition of 0% (control), 0.50%; 1.0%, 1.5% and 2.0% of mannoprotein preparations, sterilized at 121 °C for 20 min (PHCBI MLS 3751L autoclave, PHC Corporation). The media applied to the microtiter plates in a volume of 150 µL was inoculated with the inoculum of individual bacteria in a volume of 10 µL. The 24-h stationary cultures propagated in MRS medium at 37 °C (Q-CELL 240 incubator) were used as an inoculum. The inoculum was prepared by bacterial culture centrifugation in sterile tubes (4000 rpm, 5 min, Minispin Plus centrifuge, Eppendorf). The biomass pellet was then used to prepare an inoculum with a density of 0.5 McFarland in saline (Densimat, Biomerieux). The results of bacterial growth in the tested media were read using the Mulitiskan Sky apparatus (ThermoFisher Scientific) by measuring the optical density at a wavelength of 590 nm at time 0 (immediately after inoculation), after 24 h of cultivation at 37 °C (Q-CELL incubators 240). The growth intensity of the tested bacteria was determined on the basis of changes in the optical density of the cultures relating them to the changes in the control medium, which were treated as growth under optimal conditions (100%).

### Statistical analysis of the results

The results were presented based on heatmaps. The hierarchical cluster analysis was used. The heatmaps were obtained using the R platform and grouping method taking the Euclidean distance and the Ward method as a measure to illustrate the relationship between bacterial growth and the composition of the medium.

Statistical data was evaluated using the STATISTICA V.13.1 software kit (StatPoint Technologies, Inc., Warrenton, VA, USA). An analysis of variance with the one-way ANOVA method and HDS Tuckey test was carried out at the *α* = 0.05 level of significance to assess the significance of the differences. Pearson’s rank correlation beetwen nitrogen content and protein content was determined also.

## Results

### Characteristics of the obtained mannoprotein preparations

The first step of the study was to characterize isolated preparations. The obtained results confirmed their different chemical composition and structure. The results of elemental analysis differentiated the obtained mannoprotein preparations in terms of the contents of nitrogen, carbon and hydrogen (Table 1). The preparation of *Wickerhamomyces* origin mannoproteins was characterized by the lowest content of all the elements determined. Presumably, discussed sample of *Wickerhamomyces* origin could have a higher water content or contained another contamination, like inorganic salts. The tendency in nitrogen content correlated with the protein content in the preparations (correlation coefficient r = 0.99). Mannoprotein preparations isolated from the biomass of unconventional yeast *M. reukaufii* WLP 4650 and *W. anomalus* CCY 38-1-13 contained significantly less protein compared to the preparation of *S. cerevisiae* ATCC 7090 orign (Table 1). The content of total sugars was the highest in the *Metschnikowia* origin preparation. The preparation isolated from *Wickerhamomyces* yeast biomass was characterized by the lowest content of both determined components.

**Table 1:** Results of elemental analysis, protein and total sugars content in studied mannoprotein preparations

Preparation	Nitrogen	Carbon	Hydrogen	Protein	Total sugars
	Mass %			Mass %	
MP**_**Sc*	6.96 ± 0.11 a	31.90 ± 0.39 a	4.42 ± 0.06 a	42.3 ± 0.4 a**	55.7 ± 1.2 b
MP**_**Mr	6.21 ± 0.13 b	30.38 ± 0.37 b	4.12 ± 0.08 b	37.8 ± 0.3 b	60.0 ± 1.4 a
MP**_**Wa	5.41 ± 0.07 c	28.77 ± 0.34 c	3.93 ± 0.04 c	32.9 ± 0.1 c	52.8 ± 1.8 b

*MP_Sc – mannoproteins isolated from *S. cerevisiae* ATCC 7090; MP_Mr—mannoproteins isolated from *M. reukaufii* WLP 4650; MP_Wa—mannoproteins isolated from *W. anomalus* CCY 38-1-13; **a, b, c—values marked with the same letters in the column do not differ significantly at the significance level α = 0.05

The composition of amino acids associated with proteins of the three mannoprotein preparations was presented in Table 2. The tested preparations did not differ significantly in the amino acid composition. Amino acids that are acidic in nature (aspartic and glutamic acids) account for about 23% of total amino acids while base amino acids (arginine, lysine and histidine) accounted for approx. 16%. The content of amino acids hydrophobic in nature (glycine, alanine, valine, leucine, isoleucine, proline, phenylalanine and methionine) was app. 37%. The hydrophilic amino acids (aspartic acid, glutamic acid, arginine, histidine, lysine, serine and threonine) was app. 53%.

**Table 2:** Results of amino acids composition of studied mannoprotein preparations

Amino acid	Preparation		
	MP**_**Sc*	MP**_**Mr	MP**_**Wa
	Mass %		
Aspartic acid	10.9 ± 1.6 a**	10.0 ± 0.3 a	10.4 ± 1.3 a
Threonine	6.7 ± 0.9 a	7.5 ± 0.2 a	6.2 ± 1.4 a
Serine	6.4 ± 0.6 a	6.9 ± 0.1 a	6.7 ± 0,0 a
Glutamic acid	12.8 ± 1.6 a	11.9 ± 0.2 a	12.6 ± 1.6 a
Proline	5.0 ± 0.4 a	4.2 ± 0.8 a	4.5 ± 0.1 a
Glycine	6.8 ± 2.2 a	7.2 ± 1.8 a	6,8 ± 2,0 a
Alanine	7.4 ± 1.7 a	7.3 ± 1.8 a	7,0 ± 2,0 a
Valine	6.2 ± 1.3 a	6.4 ± 1.1 a	5.6 ± 1.9 a
Isoleucine	4.1 ± 0.7 a	4.9 ± 1.8 a	3.8 ± 0.4 a
Leucine	5.7 ± 0.6 a	6.2 ± 1.3 a	5.4 ± 0.3 a
Tyrosine	2.9 ± 0.6 a	2.8 ± 0.4 a	2.4 ± 0.0 a
Phenylalaine	2.6 ± 0.6 a	2.3 ± 0.1 a	2.6 ± 0.6 a
Histidine	2.4 ± 0.9 a	2.0 ± 0.4 a	1.6 ± 0.1 a
Lysine	9.5 ± 0.5 a	9.8 ± 0.0 a	9.4 ± 0.2 a
Arginine	4.3 ± 0.9 a	4.0 ± 0.3 a	5.2 ± 2.2 a
Cysteine	1.1 ± 0.2 a	1.3 ± 0.2 a	1.5 ± 0.3 a
Methionine	5.2 ± 0.4 a	5.3 ± 0.4 a	6.5 ± 1.0 a

*MP_Sc—mannoproteins isolated from *S. cerevisiae* ATCC 7090; MP_Mr—mannoproteins isolated from *M. reukaufii* WLP 4650; MP_Wa—mannoproteins isolated from *W. anomalus* CCY 38-1-13; **a—values marked with the same letter in the row do not differ significantly at the significance level α = 0.05

**Fig. 2:**
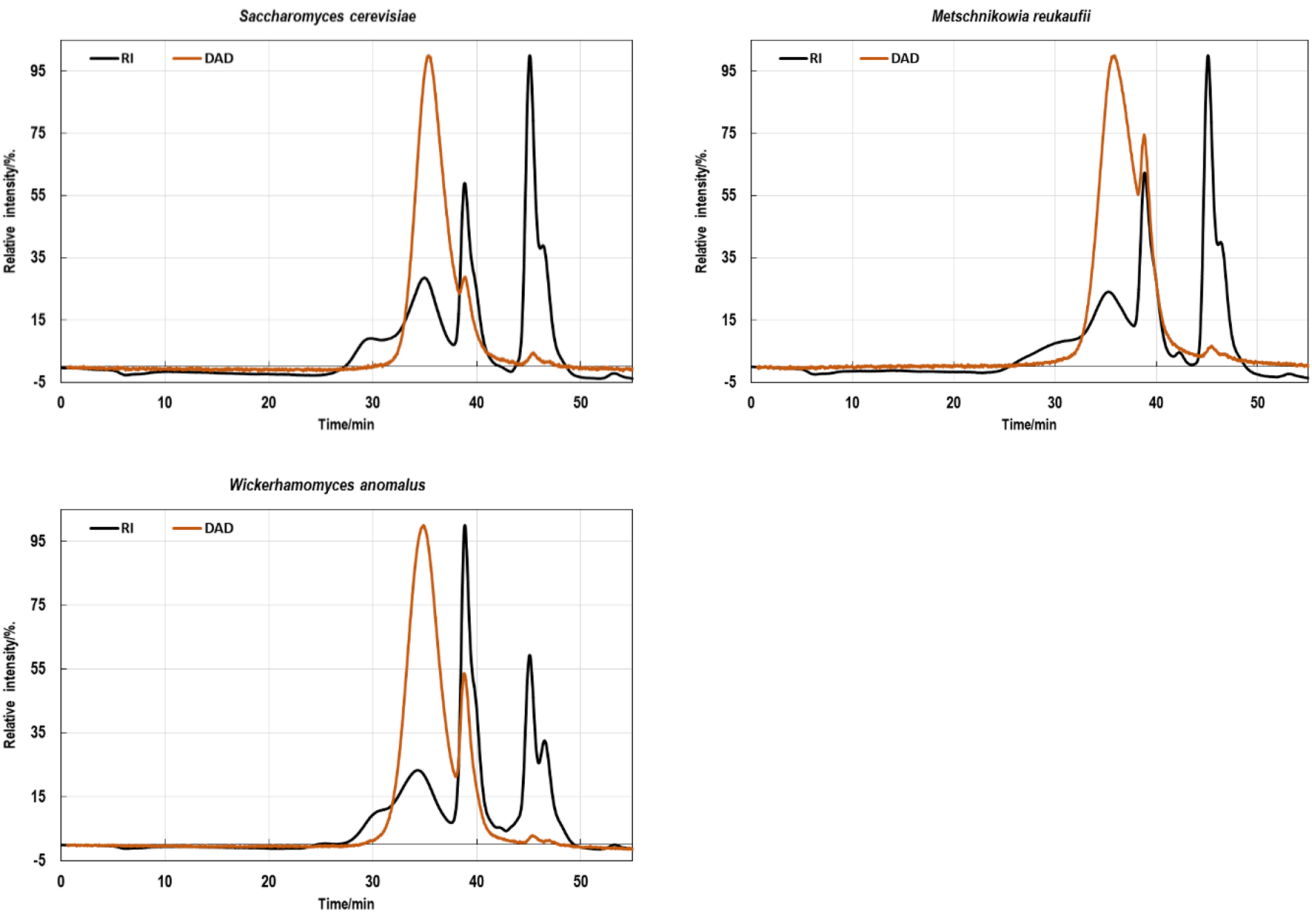
GPC-HPLC chromatograms of the mannoproteins samples. Note—time for RI and DAD detectors was synchronized

The chromatographic separation of the tested mannoprotein preparations showed that each consisted of at least three fractions with molecular weights ranging from about 1.9 to about 150 kDa, depending on the origin (Table 3, Fig. 2). Based on the analysis of chromatograms obtained using DAD detector it was concluded that the main portion of proteins was in mannoprotein molecular fraction between 13 and 20 kDa while the fractions with highest molecular weight (app. 65–150 kDa) did not contain proteins (Fig. 2).

**Table 3:** The mean molecular mass values (*M*_W_) of the main fractions identified in studied mannoprotein preparations

Preparation	Fraction		
	1	2	3
	*M*_W_/kDa		
MP_Sc*	65	14	1.9
MP_Mr	150	13	1.9
MP_Wa	84	20	1.9

*MP_Sc—mannoproteins isolated from *S. cerevisiae* ATCC 7090; MP_Mr—mannoproteins isolated from *M. reukaufii* WLP 4650; MP_Wa—mannoproteins isolated from *W. anomalus* CCY 38-1-13

**Fig. 3:**
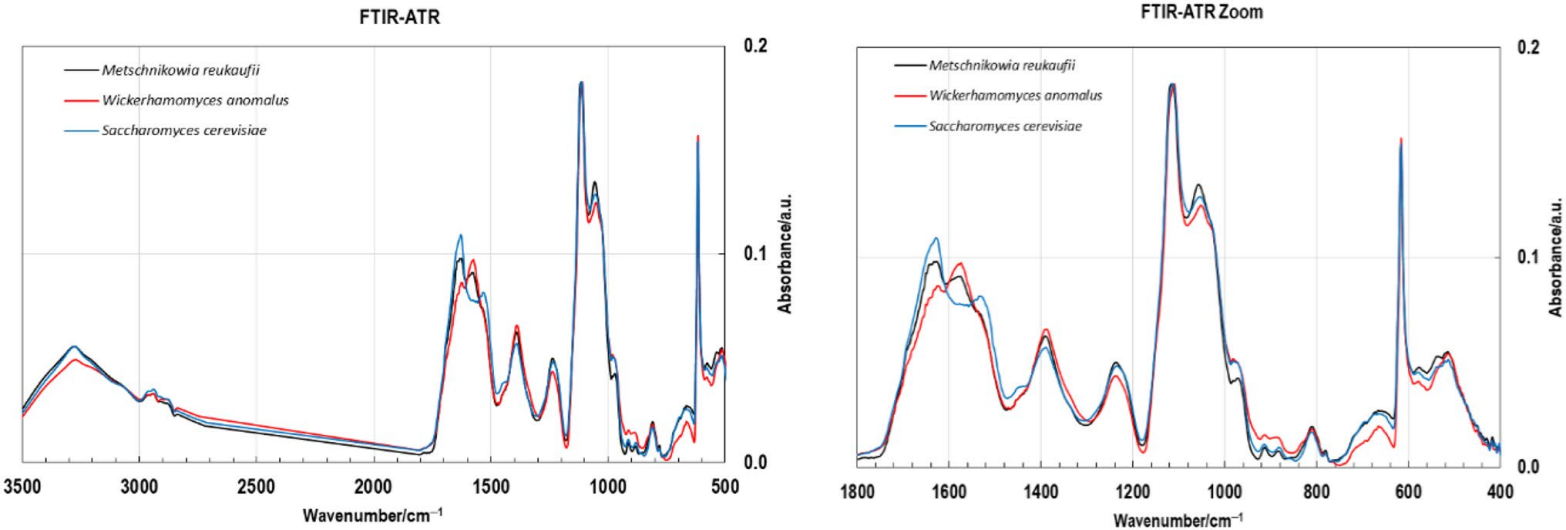
FTIR-ATR spectra of the mannoproteins

FTIR-ATR spectra of studied preparations (Fig. 3) showed the presence of greatest intensity of O–H band at a lower frequency (3272 cm^−1^). The band at 2940 cm^−1^, associated with C–H stretching (alkane), was detected for all studied preparations. Strong bands of amid I (stretching vibration of C=O at 1626–1629 cm^−1^) and amid II (banding vibration of N–H at 1534–1575 cm^−1^) indicate the presence of proteins in studied samples. This bands absorbance is proportional to N content (to protein content). Band visible at 1052 cm^−1^ was in characteristic for the stretching vibration of C–O, C–C, and ring vibration (1200–1000 cm^−1^ region) of *α*-mannan assignments. Also, the anomeric region the bands at 913–914 cm^−1^, 882–884 cm^−1^ and 810–811 cm^−1^ noted are specific for *α*-mannans. There was absent the band at 851 cm^−1^ typical for *α*-glucans (possible and often present starch contamination) and the band at 894 cm^−1^characteristic for *β*-glucans, which is the main component of yeast cell wall, however, in mannoprotein preparations it would be an impurity. There was band at 972 cm^−1^ identified and specific for mannans.

**Fig. 4:**
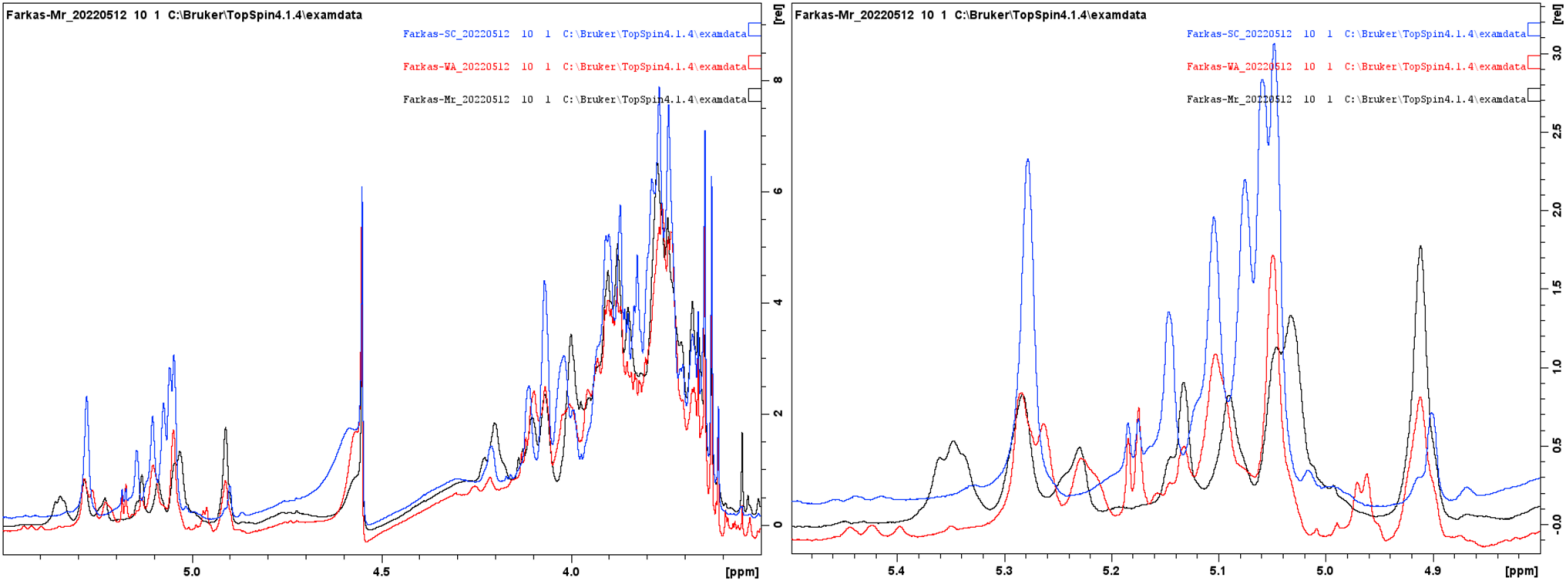
1H NMR spectra of studied preparations (on the left) and ^1^H NMR spectrum of the mannoproteins, anomeric region zoom (at the rigth); blue line—mannoproteins isolated from *S. cerevisiae* ATCC 7090; black line—mannoproteins isolated from *M. reukaufii* WLP 4650; red line—mannoproteins isolated from *W. anomalus* CCY 38-1-13

The otained ^1^H NMR spectra of studied preparations (Fig. 4) were very different from each other. Protons at 5.41–5.43 ppm were missing, indicating absence of phosphate bound mannose units. All proton anomeric signals were in *α*-region. The exact characteristics, including the structure of the isolated polymers, was not the main goal of the presented research stage. In the future, a more detailed chemical characterization of the tested preparations will be carried out. The planned subjects of further research are the sugar composition analysis, methylation analysis, TOCSY and HSQC NMR experiments, than preparation and separation of oligomers and their separate NMR spectroscopy characterization. This would allow to determine what kind of polysaccharides exactly are present in isolated preparations (mannan, glucomannan or other polysaccharide) and which fraction/s is/are important for the prebiotic or antimicrobial properties of the tested preparations.

### Antimicrobial effect of studied mannoprotein preparation

**Fig. 5:**
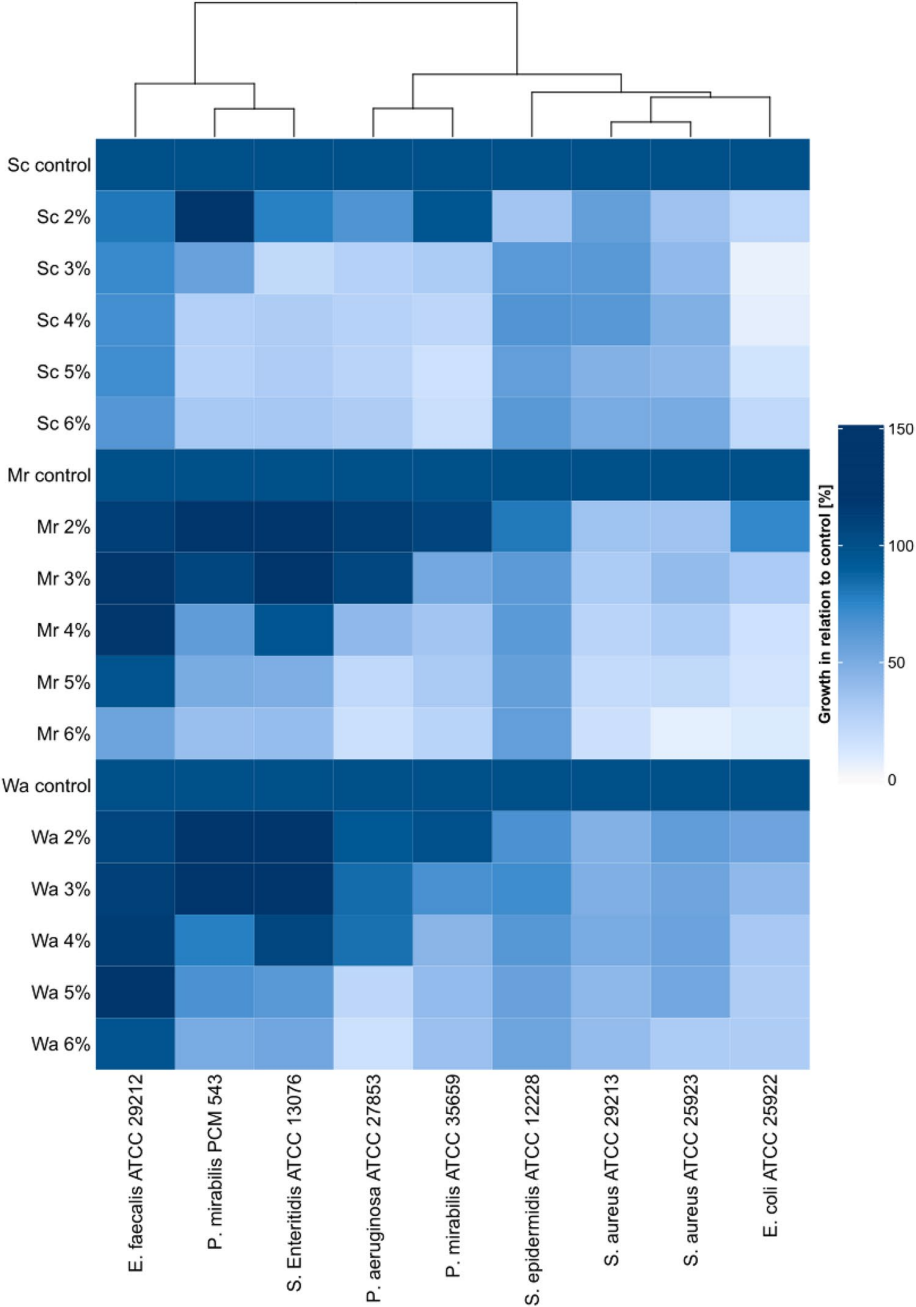
Effect of the *S. cerevisiae* ATCC 7090, *M. reukaufii* WLP 4650 and *W. anomalus* CCY 38-1-13 yeast mannoprotein preparation on the growth of selected pathogenic and saprophytic bacteria after 24 h of incubation depending on the dose of the preparation

The effect of studied mannoprotein preparations on the growth of selected pathogenic and saprophytic bacteria was determined on the basis of the following strains: *S.aureus* ATCC 25923, *S*. *aureus* ATCC 29213, *S. epidermidis* ATCC 12228, *E. faecalis* ATCC 29212, *P. aeruginosa* ATCC 27853, *P. mirabilis* ATCC 35659, *P*. *mirabilis* PCM 543, *S.* Enteritidis ATCC 13076 and *E. coli* ATCC 25922. Bacterial microcultures were carried out in Mueller–Hinton medium with the mass addition of 0% (control), 2%, 3%, 4%, 5% and 6% of mannoprotein preparations, and bacterial growth was determined on the basis of changes in the optical density of the 24-h culture in relation to the control medium. The obtained results are presented on Fig. 5.

The antimicrobial activity occured to depend on the type of preparation and its dose, as well as the type of saprophytic and pathogenic bacteria that were exposed to the preparation. The preparation of the yeast mannoprotein *S. cerevisiae* ATCC 7090 limited the growth of the *E. coli* ATCC 25922 strain to the greatest extent. In this case, the growth inhibition reached the level of approx. 77–95% depending on the concentration of the preparation in the medium (Fig. 5). Bacteria of the species *P. aeruginosa*, *P. mirabilis* and *S.* Enteritidis showed significant growth inhibition in the presence of 3–6% of the preparation. The doses between 3 and 6% of the *Saccahromyces cerevisiae* origin mannoproteins limited the growth of all studied bacteria. The 2% dose of the *S. cerevisiae* mannoprotein preparation limited the growth of the strains to a lesser extent from the genera *Salmonella* and *Pseudomonas* (by approx. 23–35%), while the bacteria *P. mirabilis* PCM 543 in the presence of the indicated dose of the preparation showed more intensive growth than in the control medium. The least sensitive to the presence of the *Saccharomyces* yeast mannoprotein preparation were Gram-positive cocci of the genera *Enterococcus* and *Staphylocoocus*, the latter being the highest.

In the case of the preparation derived from the yeast *M. reukaufii* WLP 4650, the highest growth inhibition was observed in the cultures of *S. aureus* ATCC 25923 and *E. coli* ATCC 25922 containing 6% of the preparation (93.6% and 91% inhibition, respectively)—Fig. 5. In the whole range of the concentrations used, the preparation most intensely limited the growth of *S. aureus* bacteria. Doses of 2% and 3% of the preparation stimulated the growth of the following strains: *P. aeruginosa* ATCC 27853, *P. mirabilis* ATCC 27853 and *S.* Enteritidis ATCC 13,076. In the case of concentrations of 4–6% of the preparation in the media, the growth of the indicated bacteria was significantly limited (in the range above 50%). The bacteria *E. facecalis* ATCC 29212, as well as in the presence of the preparation isolated from the yeast *Saccharomyces*, showed no sensitivity to the presence of mannoproteins of *Metschnikowia* origin. Additionally, doses of 2–4% of the discussed mannoproteins intensified the growth of the indicated bacteria. The growth of *S. epidermidis* ATCC 12228 was limited in similar extend in the presence of all studied concentrations of *M. reukaufii* WLP 4650 mannoproteins.

In the entire range of the concentrations used, the preparation obtained from the biomass of the yeast *W. anomalus* CCY 38-1-13 limited of the growth of *E. coli* ATCC 25922 to the greatest extent. Depending on the dose, the growth limitation ranged from 45.5 to 70.5%—Fig. 5. The strongest inhibitory effect of the preparation was noted at its concentration of 6% in the culture of *P. aeruginosa* strain ATCC 29212 (84.4%). The stimulating effect was observed in the case of the strain *E. faecalis* ATCC 29212 (at doses of 2–5%) and *P. mirabilis* PCM 543 (at doses of 2% and 3%), and *S.* Enteritidis ATCC 13,076 in the presence of 2–4% of the preparation. In the case of the mannoproteins of the yeast *W. anomalus* and *M. reukaufii*, it was observed that the higher concentration of the preparation intensified the inhibitory effect on the tested bacteria (except for the strain *E. faecalis* ATCC 29212), which in the case of the preparation obtained from the yeast S*. cerevisiae* was visible only in the case of Gram-negative bacteria. In the future, the mechanism of the antimicrobial activity of the tested preparations should be determined and the minimal inhibitory and bactericidal concentrations.

### Prebiotic effect of studied mannoprotein preparations

**Fig. 6:**
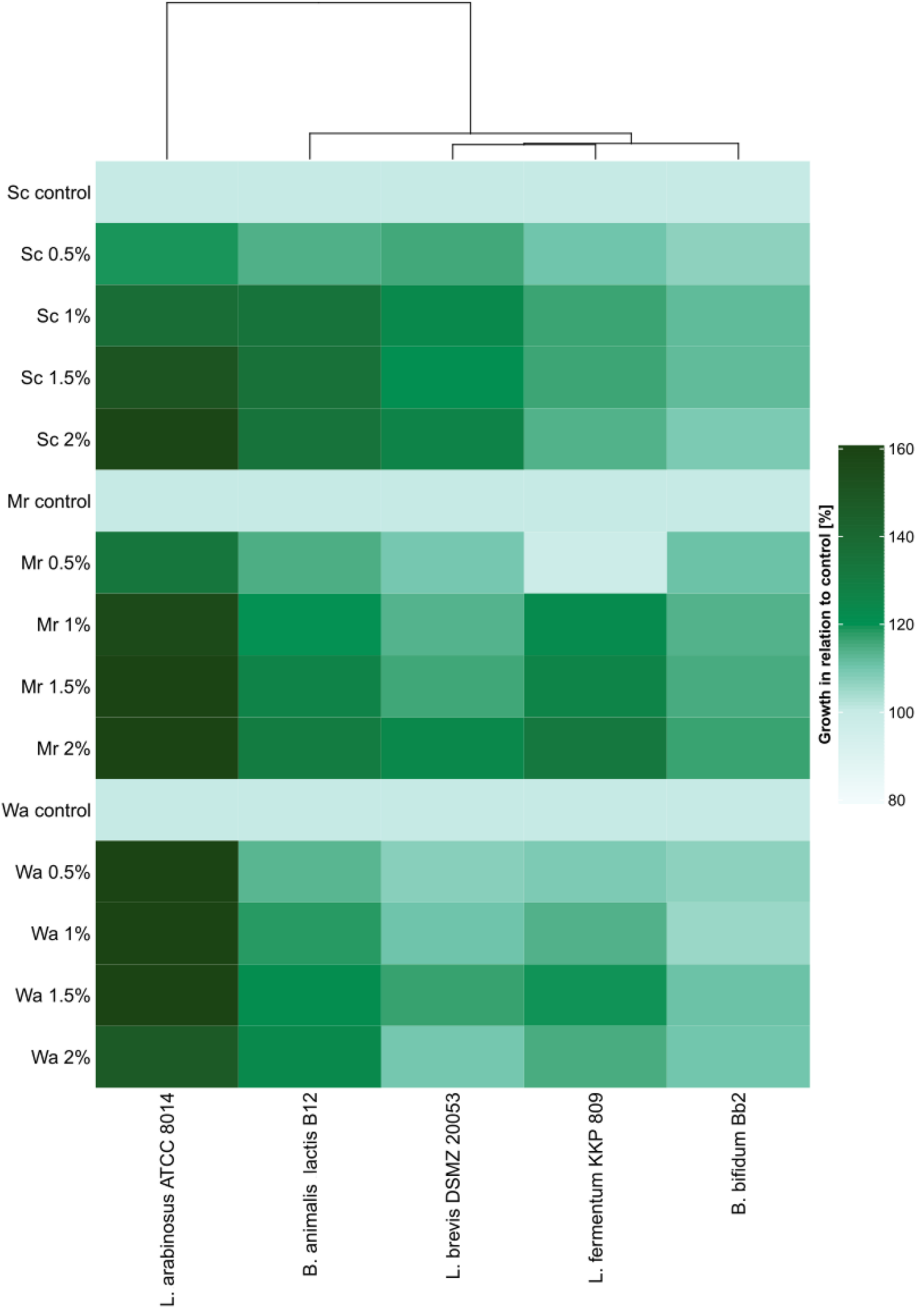
Effect of the *S. cerevisiae* ATCC 7090, *M. reukaufii* WLP 4650 and *W. anomalus* CCY 38-1-13 mannoproteins preparation on the growth of lactic acid bacteria and bifidobacteria after 24 h of incubation depending on the dose of the preparation

The prebiotic properties of the obtained mannoprotein preparations were determined for three strains of lactic bacteria: *L. arabinosus* ATCC 8014, *L. brevis* DSMZ 20053 and *L. fermentum* KKP 809 and two strains of bifidobacteria: *B. bifidum* Bb2 i *B. animalis* subsp. *lactis* B12. The MRS medium was supplemented with preparations of mannoproteins in the mass doses of 0.5%; 1.0%; 1.5% and 2.0%. The control medium was MRS without the addition of mannoproteins. The results of bacterial growth intensity in model media in relation to the control medium after 24 h of cultivation are presented on Fig. 6.

It was observed that the addition of all mannoprotein preparations in the entire range of concentrations used (0.5–2%) had a beneficial effect on the growth of lactic acid bacteria and bifidobacteria, hovewer the degree of stimulation of their growth depended on the type of preparation, its dose and the bacterial strain. In the case of the preparation obtained from the biomass of *S. cerevisiae* ATCC 7090 (Fig. 6), the most effective growth stimulation was found in the cultures of *L. arabinosus* ATCC 8014 and *B. animalis* subsp. *lactis* B12 at the concentrations of the preparation in the range of 1–2%. QueryThe growth of the *L. arabinosus* strain reached the level of approx. 159% in relation to the control culture, and *B. animalis* subsp*. lactis* approx. 135%. In the case of the remaining bacterial strains, growth was stimulated to a lesser extent (by about 7 to 26% in relation to the control), depending on the strain and dose of the preparation.

The preparation of mannoproteins isolated from the biomass of *M. reukaufii* WLP 4650 had the most beneficial effect on the growth of the *L. arabinosus* ATCC 8014 strain at the concentration of 2%, because these bacteria multiplied in such a medium more than twice as intensively (approx. 214%) compared to the control medium (Fig. 6). The addition of the preparation to the MRS medium in doses of 1–2% had a positive effect on the growth of *L. fermentum* KKP 809 and *B. animalis* subsp. *lactis* B12 (the growth more was intense by approx. 20–32% compared to the MRS medium).

Mannoproteins obtained from *W. anomalus* CCY 38-1-13 cells also had the most beneficial effect on the growth of *L. arabinosus* ATCC 8014 (Fig. 6), intensifying the growth of the indicated bacteria by approx. 50–83% depending on the dose of the preparation. In the case of the remaining strains, it only slightly intensified their growth.

It should be noted that *L. arabinosus* ATCC 8014 cells were distinguished by the most effective growth in the presence of all tested preparations, therefore it would be interesting to learn about their genetic adaptation to the use of mannoproteins as a carbon source. It is also very interesting to determine the influence of the chemical structure of a mannoprotein preparation, taking into account the structure and composition of individual fractions, on the growth of lactic acid bacteria and bifidobacteria.

## Discussion

Compared to *Saccharomyces* genera unconventional yeast may be a significantly more efficient source of mannoproteins with interesting properties (Junior et al. 2020), therefore it is worth looking for new sources of these glycoproteins. In case of (Pérez-Sotelo et al. 2005). The functional properties of yeast mannoproteins depend on their chemical characteristic (Oktaviani et al. 2021). There is relatively little information available in the literature on the relationship between the structure and composition of mannoproteins and the yeast species, as well as about the influence of mannoproteins’ characteristic on their biological activity. Presumably, for the mannoprotein-bacterial cells interactions assessed in our study, the chemical structure of the mannoproteins was the major importance, not simply the chemical composition of the preparations. This is indicated by the results of the microbiological part of the work (Figs. 5 and 6) in relations to chemical characteristic of tested preparations (Tables 1, 2, Fig. 4). Mannoproteins consist mainly with sugar residues (Quirós et al. 2011). It was the tendency observed also in our work (Table 1). Hovewer, a diversified content of sugars, protein, amino acids and ash was found, depending on the method of mannoprotein isolation, yeast and growth conditions (Kessler and Nickerson 1959; Ganan et al. 2009; Liu et al. 2011; Wan et al. 2021).

The obtained mannoprotein preparations differ in molecular weigth depending on yeast strain. This observation is consistent with the literature data but also other factors may modulate this characteristic, like yeast growth conditions and/or the isolation procedure (Franziskus and Kulicke 1999; Wan et al. 2021; Tang et al. 2022). Liu et al. (2011) indicated that chemical structure of mannoproteins extracted by hot water (procedure used in our work) was protected from chemical degradation.

The FTIR-ATR spectra of studied preparations (Fig. 3) were specific for α-mannans (da Silva Araújo et al. 2014; Galichet et al. 2001; Križková et al. 2001; Yehia et al. 2022) with no contamination with α- and β-glucans. Cosidering 1H NMR spectrum (Fig. 4) the polysaccharide fractions of studied non-conventional yeast were structurally distant from those of *Saccharomyces* or *Candida* species presented in literature, while mannoprotein obtained from *S. cerevisiae* biomass was in well compliance with published results of mannan structure (Vinogradov et al. 1998). Our results confirm observations of other studies that the structure of mannoproteins varry considerably among yeast species (Spencer and Gorin 1970; Lesage and Buseey 2006; Agboola et al. 2021). The special properties of mannoprotein unfolded, like length, type and fexibility of the branching and structure of *α*-mannoside residues in mannoproteins influence on adhesive properties of the yeast cells and as a cosequence on capability to adhere with enteropathogenic bacteria (Agboola et al. 2021). Therefore, the obtained preparations should be characterized in more detail in the future, to indicate factors responsible for their antimicrobial and prebiotic properties. In this case, it is worth determining the hydrophobicity of the preparations obtained. Cell surface hydrophobicity of yeasts affects their adhesion capacity (Masuoka and Hazen 2004). Yeasts may regulate cell surface hydrophobicity by altering the conformation of the mannoprotein fibrils (Kessler and Nickerson 1959). Modification of the acid-labile β-1,2-oligomannoside chain length was observed as the common mechanism by which yeast cells switch of the status of surface hydrophobicity. No overall difference in protein, hexose, or phosphate composition of mannoproteins extracted from hydrophobic and hydrophilic yeast cells was identified by quoted authors.

The results of our studies indicate different effect of studied mannoprotein preparations on bacteria, probably associated with the variability of cellular structures composition of tested bacteria and mannoprotein preparations of different origin. One of known srategies of the pathogenic bacteria to colonize and invade human and animal organs is to adhere and proliferate at the surface of host cells and tissues, despite defense mechanisms used by the host (Ribet and Cossart 2015). It was observed that probiotic yeasts may trapp pathogenic bacteria that express type I fimbriae on their surface by yeast cell-wall mannose-containing glycoprotein residues, preventing subsequent invasion of pathogens to the host (Pérez-Sotelo et al. 2005; Tiago et al. 2012). Binding potentials of yeast cell wall paraprobiotic preparations was observed to be bacterial strain-specific and yeast sample type-specific and higher with greater amount of mannose on yeast’s cell wall (Ganan et al. 2009; Posadas et al. 2017; Liu et al. 2021). The adhesion was not observed for *Shigella sonnei* and *Vibrio choleare*, and for Gram-positive bacteria, like *E. faecalis*, *Listeria monocytogenes*, *Bacillus cereus*, *Clostridium difficile* and *C. perfringens* (Tiago et al. 2012). Hovewer, the growth rate inhibition of the *Clostridium perfringens* was noted in the presence of yeast cell wall polymers by Santovito et al. (2019). According to Spring et al. (2000) yeast cell wall mannanooligosaccharide did not significantly reduce the number of cecal coliforms although they concentration was lower.

Schiavone et al. (2017) observed diffrences in the density and the distribution of mannoproteins at the surface of yeast cells. They indicated that abundance of a certain type of mannoproteins as important for yeast surface hydrophobicity and interactions with lectins. Yeast mannoproteins show also surfactant activity and are able to prevent and decrease initial surface adhesion of bacteria and biofilm formation (Walencka et at. 2007). No direct antibiotic activity of S*. cerevisiae* mannoprotein preparations against *S. aureus* and *Staphylocoocus epidermidis* cells was noted by Walencka et at. (2007) but mannoproteins were effective both in decreasing the initial deposition of staphylococci and in reducing the amount of biofilm formed and accelerated the detachment of mature staphylococcal biofilms. Quoted authors concluded that the anti-biofilm action of mannoproteins was due to influence on cell surface hydrophobicity.

The mannoprotein preparations used in our study were not subjected to the purification process. In the future it is worth to evaluate how the disscussed process may infuence mannoprotein antimicrobial properties. Saleh et al. (2020) determined the inhibitory effect of partially and completely purified mannoprotein fraction isolated from *S. cerevisiae* on chosen bacterial strains. After the process of mannoprotein purification by gel filtration the inhibitory activity of the preparation was increased towards eleven of studied bacterial isolates.

The results obtained (Fig. 5) present that depending on the dose and origin of the preparation and the strain of saprofitic and pathogenic bacteria the stimulation of growth was also noted. This applies in particular to the bacteria *E. faecalis* and *S.* Enteritidis. Pasada et al. (2017) observed that yeast cell wall preparations may have served as a source of carbon positively affecting the growth of *Salmonella* Typhimurium.

The observed differences in the growth efficiency of the studied strains of lactic acid bacteria and bifidobacteria in the presence of mannoprotein preparations isolated from *Saccharomyces*, *Metschnikowia* and *Wickerhamomyces* yeast cells may result from the different ability of studied bacteria to use mannoproteins as a source of nutrients (Fig. 6). α-Mannan is utilized by beneficial bacteria to multiply in the gut and is fermented into short-chain fatty acids, provide energy for intestinal cells and maintain intestinal morphology and function (Tang et al. 2022). Hovewer, it is utilized by lactic acid bacteria with different preferences (Tang et al. 2022). The specific structure of the preparations themselves and their chemical composition may affect the interactions with cell receptors and the susceptibility of mannoproteins to digestion with enzymes secreted by bacterial cells (Liu et al. 2011).

The yeast α-mannan preparation positively modulate abundances of Bacteroides, Parabacteroides, and Phascolarctobacterium decreasing the abundance of pathogenic bacteria at the same time. The prebiotic activity of yeast cell wall components was also confirmed in vivo in animal tests, reducing the colonization of the intestine track by pathogens (Fernandez et al. 2002; Baumgartner et al. 2022). Besides, yeast mannoproteins favorably influenced survival of lactic acid bacteria in simulated gastrointestinal juice and increased their adherence to Caco-2 cells (Ganan et al. 2012). It was proposed that the aggregation of lactic acid bacteria (LAB) with yeasts in gastric or intestinal juices might enhance the tolerance of LAB in intenstine track and improve adhesion specificity to Caco-2 cells (Hatoum et al. 2012).

Summarizing, the obtained results allow us to conclude that mannoproteins naturally occurring in the cell wall of both conventional (*S. cerevisiae*) and unconventional yeast (*M. reukaufii* and *W. anomalus*) have prebiotic properties that may benefit the production and enrichment of fermented food, and pressumably may also modulate intestinal microbiota. At the same time, they show antimicrobial activity, limiting the growth of undesirable microflora. Both the prebiotic and antimicrobial properties depend on the dose of the preparation, its origin and the bacterial strain. In order to thoroughly verify the obtained results, the research should be extended to include the analysis of the influence of the tested preparation on the growth of selected bacteria in the food environment and in the digestive track. In the future it is worth to evaluate how the proces of mannoprotein purification infuence their antimicrobial and prebiotic properties. A thorough structural analysis of the obtained purified preparations is necessary, as well as identification of the fractions important for prebiotic and antimicrobial activities. Considering the results obtained and the discussed mechanisms of mannoprotein activity, we assume that the adhesive properties of mannoproteins, related to the structure of *α*-mannoside residues, is crucial for limiting or promoting bacterial growth. The exact mechanism of interaction of studied preparations and different bacteria will be verified in the future.

## Data Availability

The data that support the findings of this study are available from the corresponding author upon reasonable request.

## References

[CR1] Agboola JO, Schiavone M, Øverland M, Morales-Lange B, Lagos L, Arntzen MØ, Lapeña D, Eijsink VGH, Horn SJ, Mydland LT, François JM, Mercado L, Hansen JØ (2021). Impact of down-stream processing on functional properties of yeasts and the implications on gut health of Atlantic salmon (Salmo salar). Sci Rep.

[CR2] Barreteau H, Delattre C, Michaud P (2006). Production of oligosaccharides as promising new food additive generation. Food Technol Biotechnol.

[CR3] Baumgärtner S, James J, Ellison A (2022). The supplementation of a prebiotic improves the microbial community in the gut and the skin of Atlantic salmon (*Salmo salar)*. Aquac Rep.

[CR4] Bzducha-Wróbel A, Błażejak S, Kieliszek M, Pobiega K, Falana K, Janowicz M (2018). Modification of the cell wall structure of *Saccharomyces cerevisiae* strains during cultivation on waste potato juice water and glycerol towards biosynthesis of functional polysaccharides. J Biotechnol.

[CR5] Bzducha-Wróbel A, Pobiega K, Błażejak S, Kieliszek M (2018). The scale-up cultivation of *Candida utilis* in waste potato juice water with glycerol affects biomass and *β*(1,3)/(1,6)-glucan characteristic and yield. Appl Microbiol Biotechnol.

[CR6] Calgaro M, Pandolfo M, Salvetti E, Marotta A, Larini I, Pane M, Amoruso A, Del Casale A, Vitulo N, Fiorio M, Felis GE (2021). Metabarcoding analysis of gut microbiota of healthy individuals reveals impact of probiotic and maltodextrin consumption. Benef Microbes.

[CR7] Chen YJ, Du CG, Guo YQ, Zhao YF, Aorigele C, Wang CJ, Simujide H, Aqima W, Zhang XY (2021). Antibacterial spectrum of four compounds from yeasts in koumiss. Pol J V Sci.

[CR8] Schiavone M, Déjean S, Sieczkowski N, Castex M, Dague E, François JM (2017). Integration of biochemical, biophysical and transcriptomics data for investigating the structural and nanomechanical properties of the yeast cell wall. Front Microbiol.

[CR9] da Silva Araújo VB, Ferreira de Melo AN, Gaspar Costa A, Castro-Gomez RH, Suely Madruga M, Leite de Souza E, Magnani M (2014). Followed extraction of *β*-glucan and mannoprotein from spent brewer's yeast (*Saccharomyces uvarum*) and application of the obtained mannoprotein as a stabilizer in mayonnaise. Innov Food Sci Emerg Technol.

[CR10] Fernandez F, Hinton M, Van Gils B (2002). Dietary mannan-oligosaccharides and their effect on chicken caecal microflora in relation to *Salmonella* Enteritidis colonization. Avian Pathol.

[CR11] Franziskus K, Kulicke WM (1999). Polymer analytical characterization of glucan and mannan from yeast *Saccharomyces cerevisiae*. Makromol Chem.

[CR12] Galichet A, Sockalingum GD, Belarbi A, Manfait M (2001). FTIR spectroscopic analysis of *Saccharomyces cerevisiae* cell walls: study of an anomalous strain exhibiting a pink-colored cell phenotype. FEMS Microbiol Lett.

[CR13] Ganan M, Carrascosa AV, De Pascual-Teresa S, Martinez-Rodriguez AJ (2009). Inhibition by yeast-derived mannoproteins of adherence to and invasion of Caco-2 cells by *Campylobacter jejuni*. J Food Prot.

[CR14] Ganan M, Carrascosa AV, de Pascual-Teresa S, Martinez-Rodriguez AJ (2012). Effect of mannoproteins on the growth, gastrointestinal viability, and adherence to Caco-2 cells of lactic acid bacteria. J Food Sci.

[CR15] Hatoum R, Labrie S, Fliss I (2012). Antimicrobial and probiotic properties of yeast: from fundamental to novel applications. Front Microbiol.

[CR16] Junior WJFL, Nadai C, Rolle L, da Silva GE, da Rocha Leãoe MHM, Giacomini A, Vincenzi S (2020). Influence of the mannoproteins of different strains of Starmerella bacillaris used in single and sequential fermentations on foamability, tartaric and protein stabilities of wines. Oeno One.

[CR17] Kessler G, Nickerson WJ (1959). Glucomannan-protein complexes from cell walls of yeasts. J Biol Chem.

[CR18] Klis FM, Mol P, Hellingwerf K, Brul S (2002). Dynamics of cell wall structure in *Saccharomyces cerevisiae*. FEMS Microbiol Rev.

[CR19] Križková L, Ďuračková Z, Šandula J, Sasinková V, Krajčovič J (2001). Antioxidative and antimutagenic activity of yeast cell wall mannans in vitro. Mutat Res Genet Toxicol Environ Mutagen.

[CR20] Lesage G, Bussey H (2006). Cell wall assembly in *Saccharomyces cerevisiae*. Microbiol Mol Biol Rev.

[CR21] Liu H-Z, Wang Q, He Y (2011). Immunoactivities and antineoplastic activities of *Saccharomyces cerevisiae* mannoprotein. Carbohydr Polym.

[CR22] Liu Y, Wu Q, Wu X, Algharib SA, Gong F, Hu J, Luo W, Zhou M, Pan Y, Yan Y, Wang Y (2021). Structure, preparation, modification, and bioactivities of β-glucan and mannan from yeast cell wall: a review. Int J Biol Macromol.

[CR23] Masuoka J, Hazen KC (2004). Cell wall mannan and cell surface hydrophobicity in *Candida albicans* serotype A and B strains. Infect Immun.

[CR24] Oktaviani N, Sarwono KA, Utama GL (2021). Bioconversion rice bran and cassava peel into yeasts cell walls mannoprotein as environmental friendly antioxidant. E3S Web Conf.

[CR25] Øverland M, Skrede A (2016). Yeast derived from lignocellulosic biomass as a sustainable feed resource for use in aquaculture. J Sci Food Agric.

[CR100] Pérez-Sotelo LS, Talavera-Rojas M, Monroy-Salazar HG, Lagunas-Bernabé S, Cuarón-Ibargüengoytia JA, de Montes Oca Jiménez R, Vázquez-Chagoyán JC (2005). In vitro evaluation of the binding capacity of Saccharomyces cerevisiae Sc47 to adhere to the wall of Salmonella spp. Rev Latinoam Microbiol.

[CR26] Posadas GA, Broadway PR, Thornton JA, Carroll JA, Lawrence A, Corley JR, Thompson A, Donaldson JR (2017). Yeast pro- and paraprobiotics have the capability to bind pathogenic bacteria associated with animal disease. Transl Anim Sci.

[CR27] Quirós M, Morales P, Pérez-Través L, Barcenilla JM, Gonzalez R (2011). A new methodology to determine cell wall mannoprotein content and release in wine yeasts. Food Chem.

[CR28] Ribet D, Cossart P (2015). How bacterial pathogens colonize their hosts and invade deeper tissues. Microbes Infect.

[CR29] Saleh AYA, Salman JAS, Aziz RA (2020). Study of the effect of mannoprotein extracted from *Saccharomyces cerevisiae* on some pathogenic bacteria. Eurasia J Biosci.

[CR30] Santovito E, Greco D, Marquis V, Raspoet R, D’Ascanio V, Logrieco AF, Avantaggiato G (2019). Antimicrobial activity of yeast cell wall products against *Clostridium perfringens*. Foodborne Pathog Dis.

[CR31] Spencer JFT, Gorin PAJ (1970). Systematics of the genera *Debaryomyces* and *Metschnikowia:* proton magnetic resonance spectra of their mannans as an aid in classification. Antonie Van Leeuwenhoek.

[CR32] Spring P, Wenk C, Dawson KA, Newman KE (2000). The effects of dietar mannaoligosaccharides on cecal parameters and the concentrations of enteric bacteria in the ceca of *Salmonella*-challenged broiler chicks. Poult Sci.

[CR33] Szkudzińska K, Smutniak I, Rubaj J, Korol W, Bielecka G (2017). Method validation for determination of amino acids in feed by UPLC. Accreditation Qual Assur.

[CR34] Tang N, Wang X, Yang R, Liu Z, Liu Y, Tian J, Xiao L, Li W (2022). Extraction, isolation, structural characterization and prebiotic activity of cell wall polysaccharide from *Kluyveromyces marxianus*. Carbohydr Polym.

[CR35] Tiago FCP, Martins FS, Souza ELS, Pimenta PFP, Araujo HRC, Castro IM, Branda RL, Nicoli JR (2012). Adhesion to the yeast cell surface as a mechanism for trapping pathogenic bacteria by *Saccharomyces* probiotics. J Med Microbiol.

[CR36] Valáriková J, Čížová A, Račková L, Bystrický S (2020). Anti-staphylococcal activity of quaternized mannan from the yeast *Candida albicans*. Carbohydr Polym.

[CR37] Vinogradov E, Petersen B, Bock K (1998). Structural analysis of the intact polysaccharide mannan from Saccharomyces cerevisiae yeast using 1H and 13C NMR spectroscopy at 750 MHz. Carbohydr Res.

[CR38] Walencka E, Wieckowska-Szakiel M, Rozalska S, Sadowska B, Rozalska B (2007). A surface-active agent from *Saccharomyces cerevisiae* influences staphylococcal adhesion and biofilm development. Zeitschrift Für Naturforschung C.

[CR39] Wan M, Wang M, Zhao Y, Deng H, Tan C, Lin S, Kong Y, Tong Y, Meng X (2021). Extraction of mannoprotein from *Saccharomyces cerevisiae* and analysis of its chemical composition and molecular structure. Int J Biol Macromol.

[CR40] Yehia RS, Saleh AM, Bani Ismail M, Al-Quraishy S, Al-Amri O, Abdel-Gaber R (2022). Isolation and characterization of anti-proliferative and anti-oxidative mannan from *Saccharomyces cerevisiae*. J King Saud Univ Sci.

