# Correction: Antimicrobial and prebiotic activity of mannoproteins isolated from conventional and nonconventional yeast species—the study on selected microorganisms

**DOI:** 10.1007/s11274-023-03528-0

**Published:** 2023-02-08

**Authors:** Bzducha-Wróbel Anna, Farkaš Pavol, Chraniuk Paulina, Popielarz Dominika, Synowiec Alicja, Pobiega Katarzyna, Janowicz Monika

**Affiliations:** 1 Department of Food Biotechnology and Microbiology, Institute of Food Sciences, Warsaw University of Life Sciences, Nowoursynowska 159C Street, 02-787 Warsaw, Poland; 2 Department of Immunochemistry of Glycoconjugates, Institute of Chemistry, Slovak Academy of Sciences, Dúbravská Cesta 9, 845 38 Bratislava, Slovakia; 3 Department of Food Engineering and Process Management, Institute of Food Sciences, Warsaw University of Life Sciences-SGGW, Nowoursynowska Str 159C, 02-776 Warsaw, Poland

**Correction: World Journal of Microbiology and Biotechnology (2022) 38:256** 10.1007/s11274-022-03448-5

Following publication of the original article [1], the author reported an error in processing given name and family name of the authors. The correct given name and family name of the authors are:

Given name: Anna, Family name: Bzducha Wróbel

Given name: Pavol, Family name: Farkaš

Given name: Paulina, Family name: Chraniuk

Given name: Dominika, Family name: Popielarz

Given name: Alicja, Family name: Synowiec

Given name: Katarzyna, Family name: Pobiega

Given name: Monika, Family name: Janowicz
